# Supplementary material for: Self-driving microscopy detects the onset of protein aggregation and enables intelligent Brillouin imaging
Source: Nat Commun. 2025 Jul 24;16:6699. doi: 10.1038/s41467-025-60912-0 (PMC12289965; doi:10.1038/s41467-025-60912-0)
Supplement: Supplementary file 1 — Supplementary Information [file 41467_2025_60912_MOESM1_ESM.pdf]

Supplementary Information for:

Self-Driving Microscopy Detects the Onset of Protein Aggregation and Enables Intelligent Brillouin Imaging

Contents:

Supplementary Figure 1: Schematic of the microscope setup used in this work

Supplementary Figure 2: Schematic of the neural network architectures used in this work

Supplementary Figure 3: Classification matrix of the IC-LINA model

Supplementary Figure 4: Graphical user interface of our SDM software

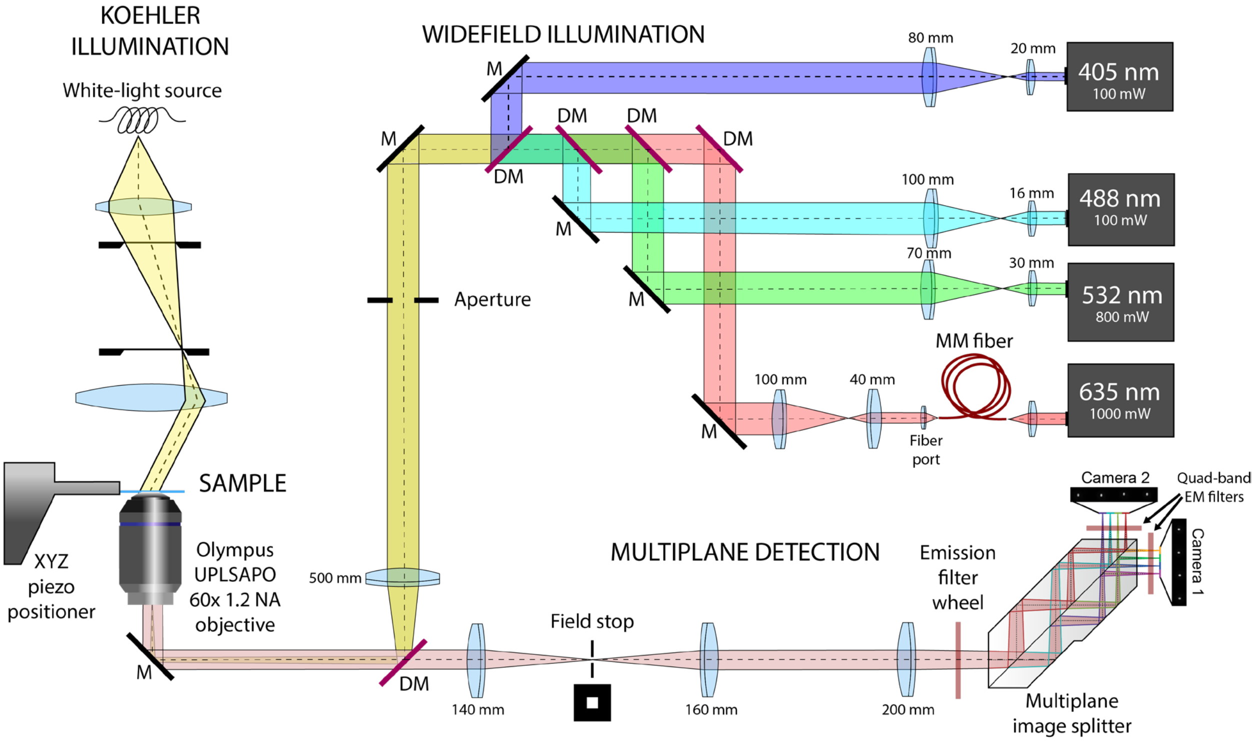

Supplementary Figure 1: Schematic of the microscope setup used in this work. M – mirror, DM – dichroic mirror, MM – multi-mode, EM – emission. Adapted from work by Navikas and colleagues[1]. First developed and described in work by Descloux and colleagues[2].

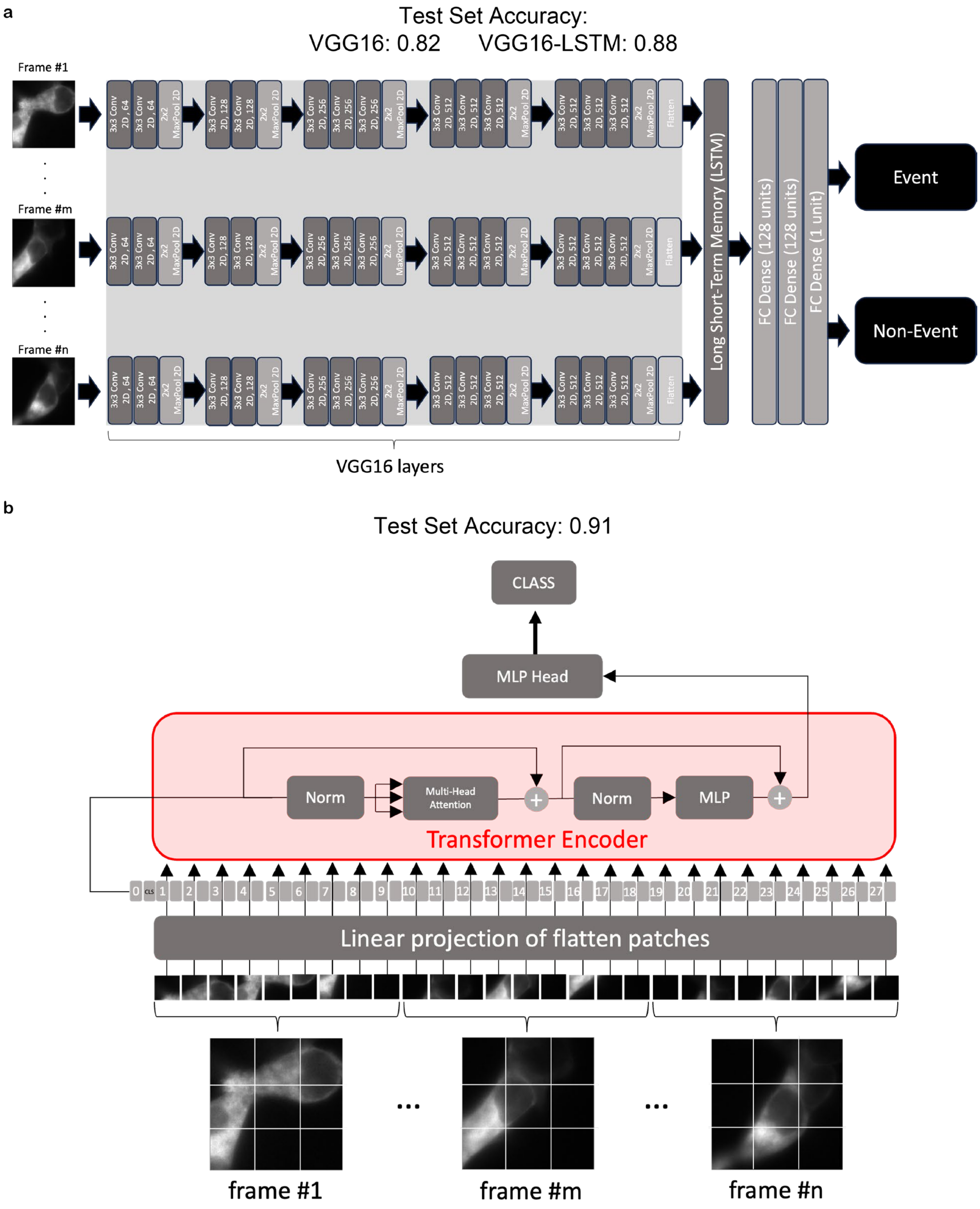

Supplementary Figure 2: Schematic of the neural network architectures used in this work. (a) VGG16 and VGG16-LSTM. (b)

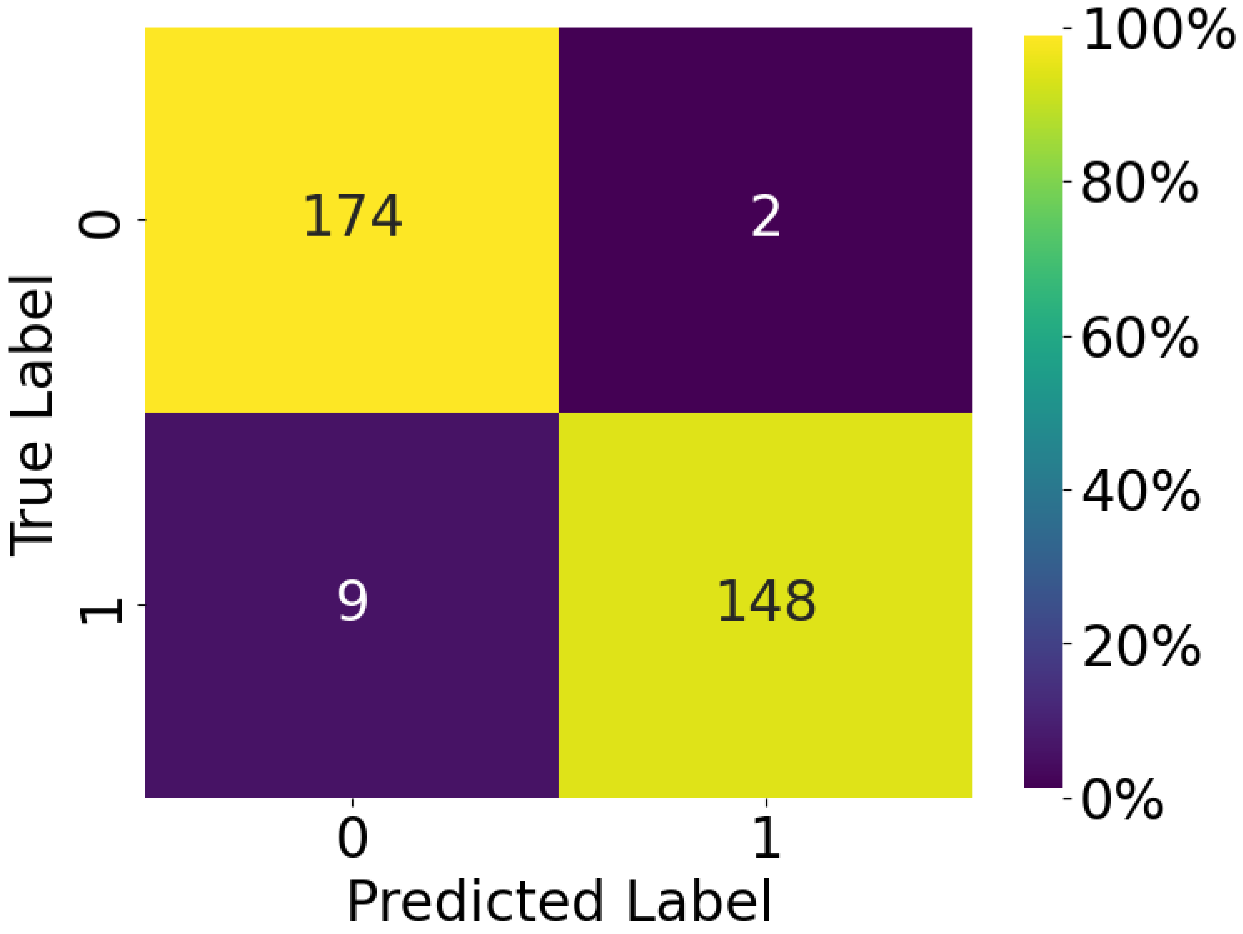

Supplementary Figure 3: Classification matrix of the IC-LINA model. Performance metrics: precision = 0.99, recall = 0.94, F1 score = 0.96, accuracy = 0.97.

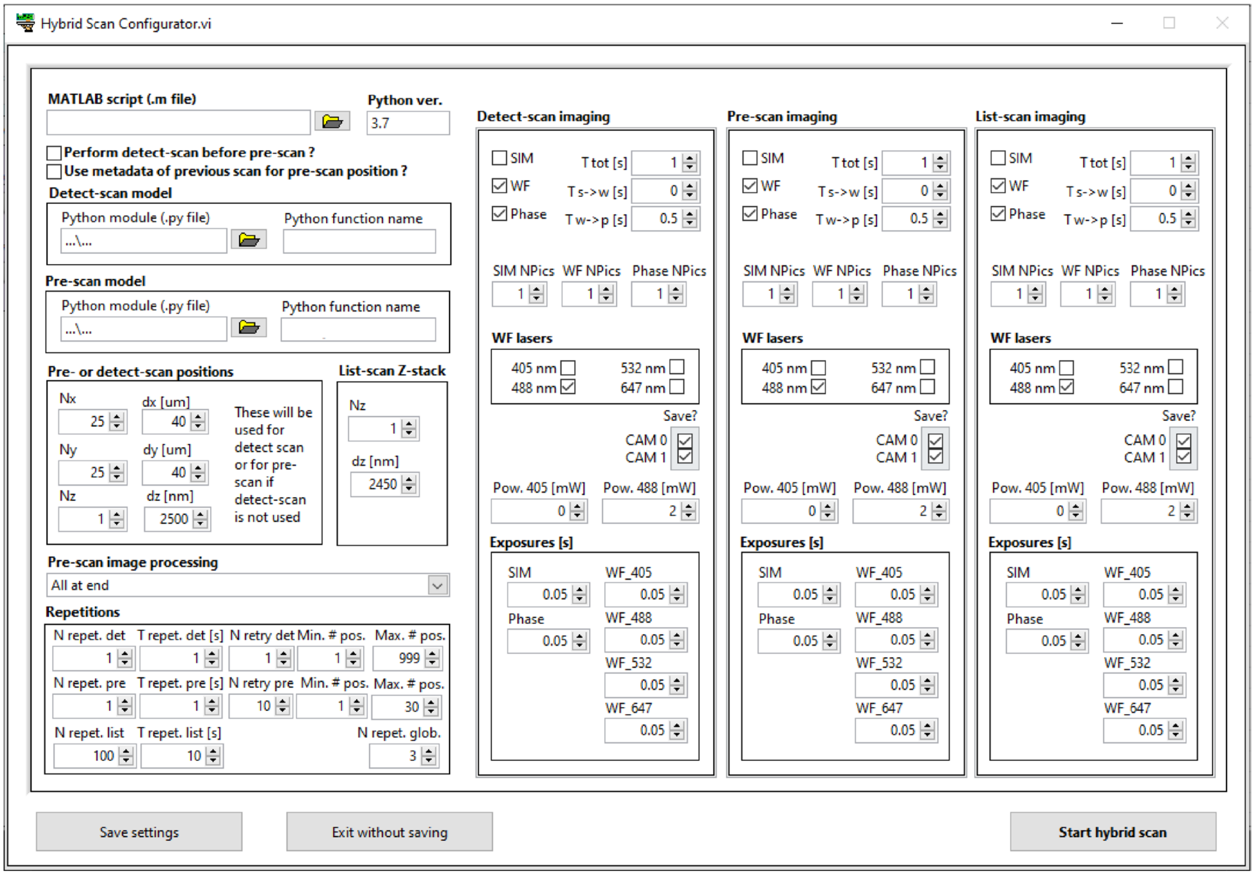

Supplementary Figure 4: Graphical user interface of our SDM software. A variety of parameters can be set by the user for both the initial and optimized scans, such as imaging modalities, exposure times, laser powers, z-stack settings, and time-lapse settings..

References

1. Navikas, V. *et al.* Correlative 3D microscopy of single cells using super-resolution and scanning ion-conductance microscopy. *Nat Commun* **12**, 4565 (2021).
2. Descloux, A. *et al.* Combined multi-plane phase retrieval and super-resolution optical fluctuation imaging for 4D cell microscopy. *Nature Photonics* **12**, 165-172, (2018).
